# Exopolysaccharide Production by *Sinorhizobium fredii* HH103 Is Repressed by Genistein in a NodD1-Dependent Manner

**DOI:** 10.1371/journal.pone.0160499

**Published:** 2016-08-03

**Authors:** Sebastián Acosta-Jurado, Pilar Navarro-Gómez, Piedad del Socorro Murdoch, Juan-Carlos Crespo-Rivas, Shi Jie, Lidia Cuesta-Berrio, José-Enrique Ruiz-Sainz, Miguel-Ángel Rodríguez-Carvajal, José-María Vinardell

**Affiliations:** 1 Departamento de Microbiología, Facultad de Biología, Universidad de Sevilla, Sevilla, Spain; 2 Departamento de Bioquímica Vegetal y Biología Molecular, Facultad de Biología, Universidad de Sevilla, Sevilla, Spain; 3 Departamento of Química Orgánica, Facultad de Química, Universidad de Sevilla, Sevilla, Spain; Estacion Experimental del Zaidin—CSIC, SPAIN

## Abstract

In the rhizobia-legume symbiotic interaction, bacterial surface polysaccharides, such as exopolysaccharide (EPS), lipopolysaccharide (LPS), K-antigen polysaccharide (KPS) or cyclic glucans (CG), appear to play crucial roles either acting as signals required for the progression of the interaction and/or preventing host defence mechanisms. The symbiotic significance of each of these polysaccharides varies depending on the specific rhizobia-legume couple. In this work we show that the production of exopolysaccharide by *Sinorhizobium fredii* HH103, but not by other *S*. *fredii* strains such as USDA257 or NGR234, is repressed by *nod* gene inducing flavonoids such as genistein and that this repression is dependent on the presence of a functional NodD1 protein. In agreement with the importance of EPS for bacterial biofilms, this reduced EPS production upon treatment with flavonoids correlates with decreased biofilm formation ability. By using quantitative RT-PCR analysis we show that expression of the *exoY2* and *exoK* genes is repressed in late stationary cultures of *S*. *fredii* HH103 upon treatment with genistein. Results presented in this work show that in *S*. *fredii* HH103 EPS production is regulated just in the opposite way than other bacterial signals such as Nod factors and type 3 secreted effectors: it is repressed by flavonoids and NodD1 and enhanced by the *nod* repressor NolR. These results are in agreement with our previous observations showing that lack of EPS production by *S*. *fredii* HH103 is not only non-detrimental but even beneficial for symbiosis with soybean.

## Introduction

Rhizobia are soil α- and β-proteobacteria able to establish a nitrogen-fixing symbiosis with legumes. In this interaction, rhizobia induce the formation of new plant organs, called nodules, on legume roots. Inside nodule cells, rhizobia differentiate into nitrogen fixing bacteroids which provide the plant with ammonia and receive in turns organic acids [1].

The nodulation process relies on a complex molecular dialogue between plant and bacteria [2–4]. The first step is the secretion of phenolic compounds called flavonoids by plant roots. The bacterial protein NodD, upon interaction with appropriate flavonoids, binds to conserved DNA sequences, called *nod* boxes, and induces the expression of bacterial *nod* genes, responsible for the synthesis and secretion of molecular signals known as Nod factors. In several rhizobial species, such as *Sinorhizobium meliloti* and *S*. *fredii*, fine-tuning modulation of *nod* gene expression is achieved by the participation of NolR, a transcriptional repressor protein [5, 6]. Recognition of appropriate Nod factors by LysM plant receptors induces early events of the nodulation process such as root hair curling and nodule primordium formation [7].

In addition to Nod factors, other bacterial molecules are required for a successful interaction [2–4]. Several rhizobia (including *S*. *fredii* strains) deliver effector proteins into plant cells through a symbiotic type 3 secretion system in a process that is also dependent on NodD and plant flavonoids [8]. These rhizobial effectors manipulate host cells in order to suppress defensive responses against rhizobia and to promote symbiosis-related processes.

In addition, several rhizobial surface polysaccharides have been demonstrated to play important roles in symbiosis, acting as signals molecules and/or preventing plant defense responses [2, 3, 9]: cyclic glucans (CG), which are cycled polymers of glucose located on the periplasm, lipopolysaccharide (LPS) and K-antigen capsular polysaccharide (KPS), which are constituents of the outer leaf of the external membrane, and exopolysaccharides (EPS), which are acidic polysaccharides located out of the cell with little or no cell association. With the exception of CG, which have been proven to be essential in all rhizobia-legume symbiosis tested so far, the relevance of these polysaccharides depends on the specific rhizobia-legume interaction analyzed [3, 10].

EPS provide protection against different stresses such as desiccation or the presence of antimicrobial compounds and have a role in attachment to surfaces and in biofilm formation [9, 11]. EPS produced by rhizobia are highly variable but mainly contain common monosaccharides such as d-glucose, d-galactose, d-mannose, as well as d-glucuronic and d-galacturonic-acids. In addition, often several non-carbohydrate substituents, such as *O*-acetyl groups or ketal-linked pyruvate and succinyl half ester groups, are also found. Very recently we have reported the structure of the *S*. *fredii* HH103 EPS, which is composed of glucose, galactose, glucuronic acid, pyruvic acid (5:2:2:1) and is partially acetylated [12]. Rhizobial EPS is essential in several interactions with legumes forming indeterminate nodules, such as *Sinorhizobium meliloti*-*Medicago* or *Rhizobium leguminosarum* bv. *trifolii*-*Trifolium* [11 and references therein], but this is not always the case. Thus, an *exoA* (EPS^-^) mutant of *S*. *fredii* HH103 is slightly impaired but still effective in its interaction with *Glycyrrhiza uralensis* or *Cajanus cajan* [10, 13]. In addition, this *S*. *fredii* HH103 *exoA* mutant is fully effective with *Glycine max* (soybean) or *Vigna unguiculata* [13, 14], two legumes forming determinate nodules, and it even shows increased competitiveness to nodulate soybean [12].

Several reports indicate that *nod* gene inducing flavonoids, such as apigenin or genistein, can affect the production of *S*. *fredii* surface polysaccharides. Thus, in strain NGR234 the presence of apigenin reduces the amount of KPS produced and elicits the synthesis of a novel kind of LPS rich in rhamnose in a NodD-dependent manner [15, 16]. None of these changes have been found in *S*. *fredii* HH103 [14, 17] or, to our knowledge, in other *S*. *fredii* strains. In 1992, Dunn et al. [18] reported that genistein provokes alterations in the molecular mass and the composition of *S*. *fredii* USDA193 EPS. More recently, we reported that both the presence of this flavonoid and the inactivation of *nolR* results in a non-mucoid phenotype in *S*. *fredii* HH103 [6].

In this work we have further investigated the relation between the *nod* regulatory network and EPS production in *S*. *fredii* HH103. We show that the effect of flavonoids on EPS production is related to their *nod* gene induction ability and, moreover, that it is absolutely dependent on NodD1. The non-mucoid phenotype is due to a decrease in EPS production and not to structural changes. This reduced EPS production is, at least partially, related to a decrease in the level of transcripts of the *exoY2* and *exoK* genes in stationary phase cultures, although the exact way in which this expression is modulated remains to be elucidated.

## Material and Methods

### Microbiological techniques

*Sinorhizobium fredii* strains HH103 Rif^R^ [19], SVQ116 (= HH103 Rif^R^ *nodA*::Tn*5*-B20, [20]), SVQ530 (= HH103 Rif^R^ *exoA*::*lacZ*Δp::Gm^R^ [13]), SVQ502 (= HH103 Rif^R^ *nodD1*::*lacZ*Δp::Gm^R^ [21]), SVQ548 (= HH103 Rif^R^ *nolR*::*lacZ*Δp::Gm^R^, this work), and SVQ554 (= HH103 Rif^R^ *nodD2*::*lacZ*Δp::Gm^R^, this work) were grown at 28°C on TY medium [22], yeast extract/mannitol (YM) medium [23] or MGM medium [24]. Plasmids pMUS296 and pMUS746 are pMP92 derivatives containing the *nodD1* and the *nodD2* genes of *S*. *fredii* HH103, respectively [21, 25]). The *nolR* and *nodD2* mutant derivatives of HH103 used in this work were constructed by homogenotization of a copy of the gene truncated by the *lacZ*Δp-Gm^R^ cassette [26] following methodologies previously used by our group [10, 13, 14, 21]. *S*. *fredii* strains USDA257 [27] and NGR234 [28] and *S*. *meliloti* Rm1021 [29] were cultured on YM medium. *Escherichia coli* was cultured on Luria-Bertani (LB) medium [30] at 37°C. When required, the media were supplemented with the appropriate antibiotics as described by Vinardell et al. [21]. Flavonoids were dissolved in ethanol at a concentration of 1 mg/mL and used at 1 μg/mL. Plasmids were transferred from *E*. *coli* to rhizobia by triparental mating by using pRK2013 as the helper plasmid [31]. The Optical Density (OD) of bacterial cultures was determined by using a Pharmacia LKB Novaspec II spectrophotometer.

For analysis of EPS production in solid media, 20 μl droplets of YMB-grown early log (OD_600_ = 0.4) cultures were placed onto YMA plates supplemented with ethanol or flavonoids (flavone, genistein, or luteolin), incubated at 28°C for 96 hours and photographed. For EPS quantification, bacterial cultures were grown in YM for 96 hours at 28°C (OD_600_ = 1.2–1.3) under shaking conditions. Cells were removed by centrifugation (20000 *g*, 15 min) and total carbohydrate amounts of the EPS-containing supernatants were determined using the anthrone-H_2_SO_4_ method, which measures the total glucose equivalents content in a given sample, as previously described [32]. Four independent experiments in duplicate were carried out. Assays for biofilm formation on plastic surfaces were carried out as described by Rodríguez-Navarro et al. [12]. Data presented are the media of at least three independent experiments performed in duplicate; in each experiment, at least 12 wells for each treatment were measured.

### Molecular techniques

Recombinant DNA techniques were performed according to the general protocols of Sambrook et al. [30]. PCR amplifications and hybridisations were performed as previously described [6]. For measuring expression of the *exoA*, *exoK*, *exoY2* and *exoA* genes, quantitative RT-PCR experiments were performed by using primer pairs rt-exoA-F2/R2, rt-exoK-F/R, exoY2rt-F/R, and qnodA-F/R respectively. The *S*. *fredii* HH103 16S rRNA was used as an internal control to normalize gene expression (primer pair rt-16S-F2/R2). All primer pairs used are shown in S1 Table. Total RNA was isolated using the High Pure RNA Isolation Kit (Roche) and RNase Free DNA Set (Qiagen) according to the manufacturer’s instructions. This (DNA free) RNA was reverse transcribed to cDNA by using PrimeScript RT reagent Kit with gDNA Eraser (Takara). Quantitative PCR was performed using a LightCycler 480 (Roche, Switzerland) with the following conditions: 95°C, 10 min; 95°C, 30 s; 50°C, 30 s; 72°C, 20 s; forty cycles, followed by the melting curve profile from 60 to 95°C to verify the specificity of the reaction. The fold changes of three biological samples with three technical replicates in each condition were obtained using the ΔΔCt method.

### Statistical analyses

For each strain and condition, the capacity of biofilm formation, the amount of glucose equivalents produced and the expression level of *nodA* and *exo* genes were compared to those of the wild type strain grown in the absence of genistein by using the Mann-Whitney non-parametrical test.

### Chemical analyses of EPS

For isolation of bacterial EPS, culture media were concentrated up to 20% of the initial volume on a rotary evaporator and three volumes of cold ethanol were added. After 24 h at 4°C, the resulting precipitates were separated by centrifugation, redissolved in water and purified by dialysis against distilled water. Finally, the solutions were concentrated and freeze dried. For NMR experiments, 1–5 mg of samples were deuterium-exchanged several times by freeze-drying from D_2_O and then examined in solution (1–5 mg/750 mL of 99.96% D_2_O). Spectra were recorded at 353 K on a Bruker AV500 spectrometer operating at 500.20 MHz (^1^H). Chemical shifts are given in ppm, using the HDO signal (4.22 ppm at 353 K) as reference [33].

## Results

### *Sinorhizobium fredii* HH103 EPS production is regulated by *nod* gene inducing flavonoids

Previous results of our research group had shown that the mucoidy of *S*. *fredii* HH103 in YM medium is negatively affected by the presence of genistein, a flavonoid present in soybean root exudates that is an effective *nod* gene inducer of this strain [6]. This result prompted us to investigate whether this effect also took place in other *S*. *fredii* strains. The ineffective *nod* gene inducer flavone [21] and ethanol (the organic solvent used to dissolve flavonoids) were used as negative controls of the effect of genistein. In addition to *S*. *fredii* strains, *S*. *meliloti* Rm1021 was also included in this experiment since it has been reported that flavonoids has a slight inducer effect on EPS I production in this strain [34]. For this reason, in addition to genistein, we also investigated the effect of luteolin, a *Medicago sativa* root-exuded flavonoid that is an effective *nod* gene inducer for both *S*. *meliloti* and *S*. *fredii* [21, 35].

**Fig 1:**
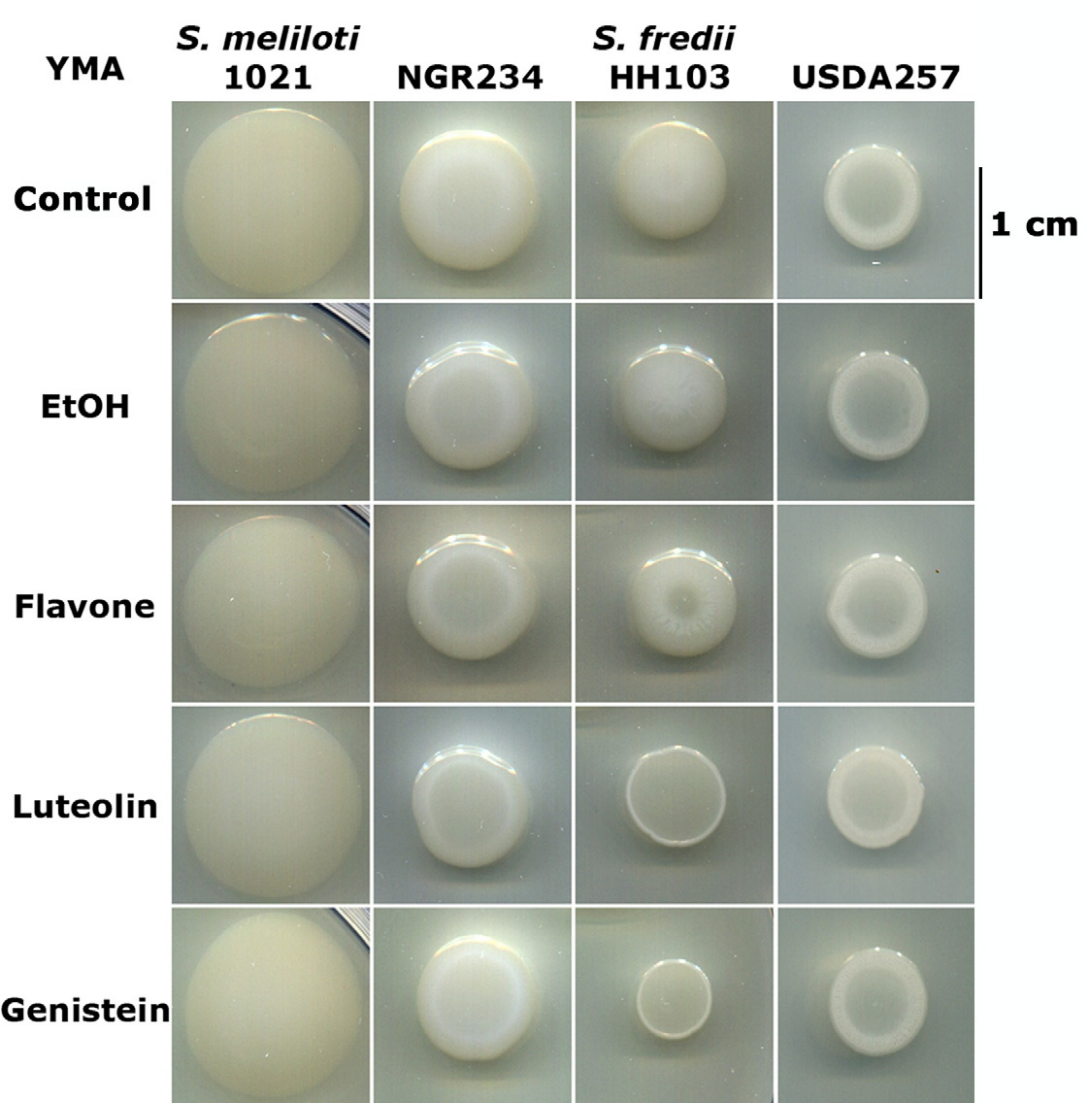
Mucoidy of different sinorhizobial strains in YMA medium in the presence or absence of flavonoids. Compounds used for supplementing YMA medium are indicated on the left.

Results presented in Fig 1 indicate that, among the different *S*. *fredii* strains tested, the flavonoid effect on EPS production appears to be specific for HH103. Thus, *S*. *fredii* HH103 presented a rough appearance when grown in YMA plates supplemented with genistein or luteolin, whereas the mucoidy of strains USDA257 and NGR234 was apparently not affected by the presence of these flavonoids. The slight positive effect of luteolin on EPS I production by Rm1021 could not be noticed by this approach. The appearance of *S*. *fredii* HH103 was not altered in YMA plates supplemented with either ethanol or flavone, indicating that neither the organic solvent used to dissolve flavonoids nor flavonoids ineffective as *nod* gene inducers affect *S*. *fredii* HH103 mucoidy.

### Flavonoid-mediated regulation of *S*. *fredii* HH103 EPS depends on the regulatory genes *nodD1* and *nolR*

**Fig 2:**
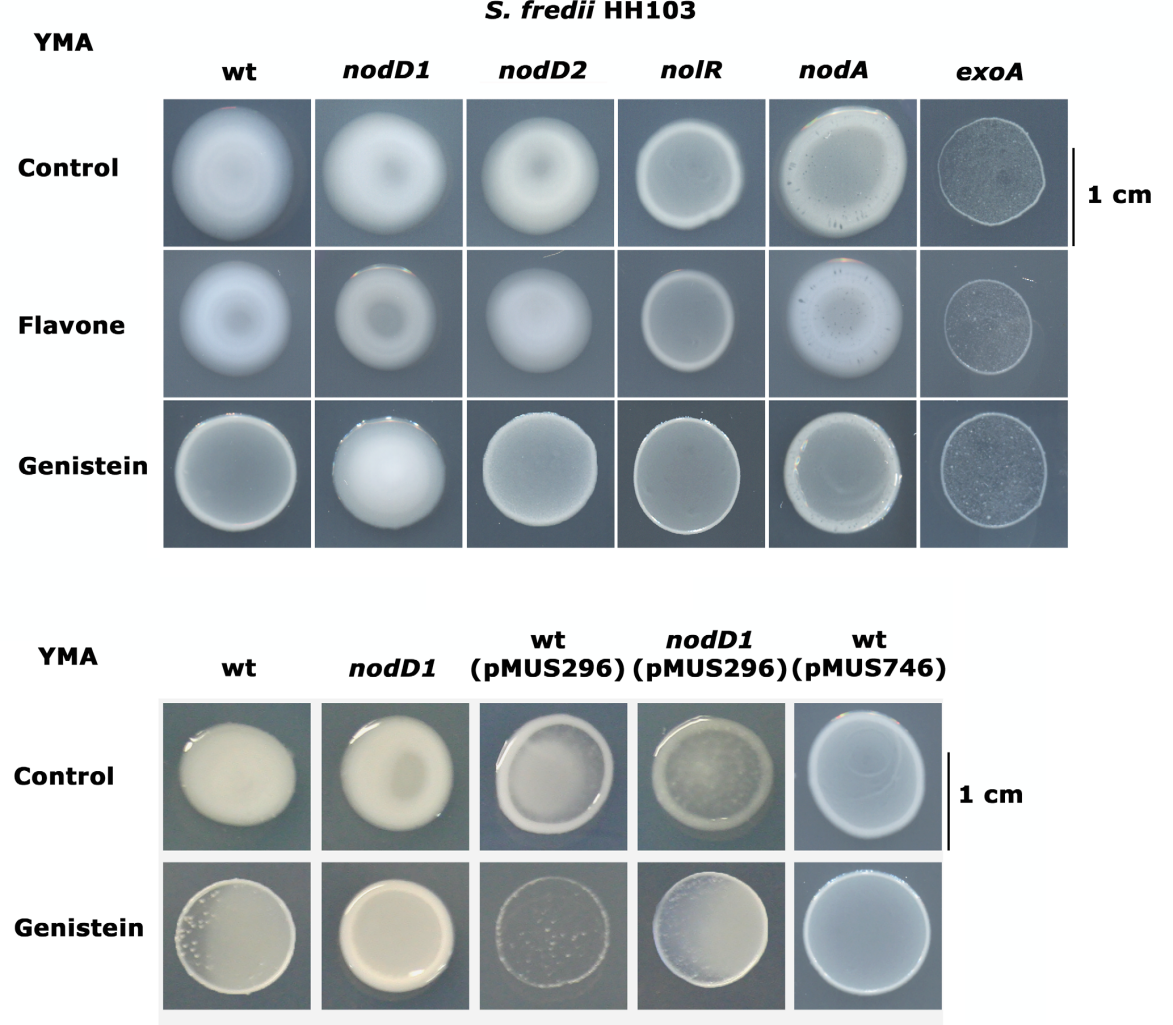
Mucoidy of *S*. *fredii* HH103 (wt) and different mutant derivatives in YMA in the absence or presence of flavone or genistein. The gene affected by the mutation in each strain is indicated on the top of each column. Plasmids pMUS296 and pMUS746 harbor wild-type copies of HH103 *nodD1* and *nodD2* respectively. Compounds used for supplementing YMA medium are indicated on the left. Control denotes absence of flavonoids.

Since only those flavonoids effective as *nod* inducers have a negative effect on *S*. *fredii* HH103 EPS production, we decided to investigate whether there is a connection between the *nod* regulon and EPS production in *S*. *fredii* HH103. For this purpose we analyzed the effect of genistein on the mucoidy of the wild type strain as well as of HH103 derivatives affected in *nodA* (unable to produce Nod factors) and in different *nod* regulatory genes present in this strain [6, 17, 21, 36]: *nodD1*, *nodD2*, and *nolR* (Fig 2). Strain SVQ530, an HH103 *exoA* derivative defective in EPS production [12], was employed as negative control for EPS production.

As expected, regardless the presence or absence of flavonoids, HH103 *exoA* showed a rough appearance. The presence of genistein, but not that of flavone, led to a decrease in mucoidy in the wild type strain and in its *nodD2* and *nodA* derivatives. Interestingly, in the absence of flavonoids or in the presence of flavone, the appearance of the *nodA* derivative was different to that of the wild type since disruptions in the mucoidy could be observed in the former strain. As previously described [6], lack of NolR led to a less mucoid phenotype of *S*. *fredii* HH103 even in the absence of flavonoids, although this phenotype was more evident in the presence of genistein. In the *nodD1* mutant background the presence of genistein did not affect HH103 mucoidy. Consistently, introduction of the wild-type *nodD1* gene (harboured by plasmid pMUS296) restored the negative effect of genistein on mucoidy in the *nodD1* mutant and even reduced those of the wild type strain and its *nodD1* mutant in the absence of genistein. These results indicate that the genistein-mediated repression of HH103 mucoidy involves the participation of NodD1. However, introduction of extra copies of *nodD2* (carried by plasmid pMUS756) also reduced mucoidy in the wild type strain in the absence of flavonoids, suggesting that, when in overdose, NodD2 could mimic the mucoidy repression exerted by NodD1.

**Fig 3:**
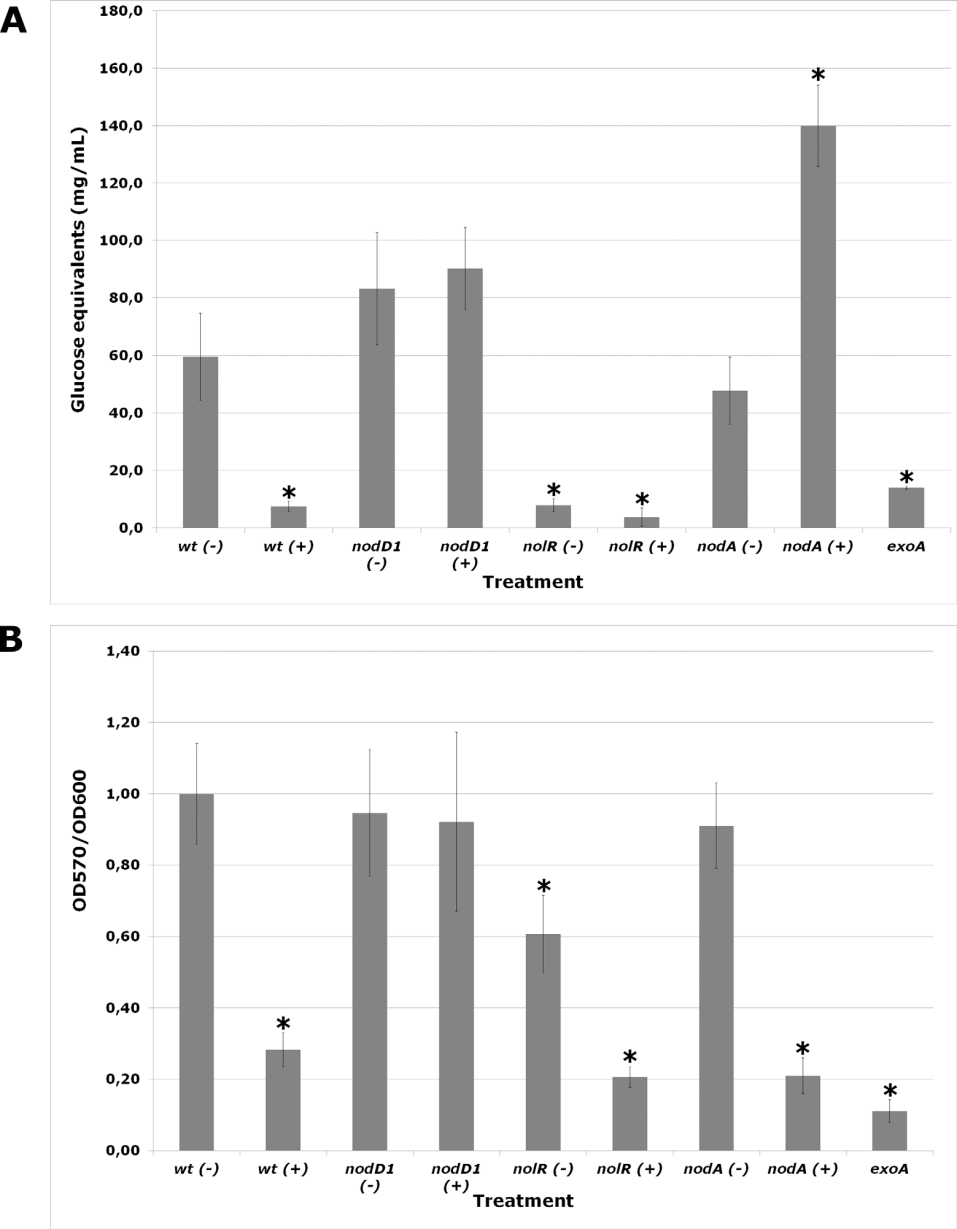
**Glucose equivalents in the extracellular milieu (A) and biofilm formation ability (B) of *S*. *fredii* HH103 (wt) and its *nodA*, *nodD1*, *nolR* and *exoA* mutant derivatives grown in the presence (+) or absence (-) of genistein.** The presence of an asterisk denotes a significant difference at the 0.01% level with respect to wt (-).

Production of EPS by *S*. *fredii* HH103 and its *nodA*, *nodD1*, and *nolR* mutant derivatives in the absence or presence of genistein was further investigated by quantification of the amount of glucose equivalents found in the extracellular milieu of cultures of these strains in YM broth (YMB) (Fig 3A). The HH103 *exoA* derivative was used as a negative control for production of EPS. The low levels of glucose equivalents detected in HH103 *exoA* might correspond to other polysaccharides present in culture supernatants such as cyclic glucans. The presence of genistein reduced about 10-fold the amount of glucose equivalents produced by HH103 (to levels similar to that of the *exoA* mutant) but did not affect that of the HH103 *nodD1* derivative. In addition, the HH103 *nolR* derivative clearly produced less amount of glucose equivalents than the wild type strain, regardless the presence or absence of genistein. In the absence of flavonoids, the *nodA* derivative of HH103 produced similar amounts of glucose equivalents when compared to the wild type strain. Surprisingly, when genistein was present, the amount of glucose equivalents present in the extracellular milieu of HH103 *nodA* was significantly enhanced in comparison to those of the non-induced cultures of both the wild-type and the *nodA* mutant strains.

In *S*. *fredii* HH103 EPS has been shown to be essential for biofilm formation ability onto plastic surfaces [12]. Because of this, we have investigated whether this bacterial capacity is also affected by the presence of genistein. The *exoA* derivative of HH103 was employed as negative control for biofilm formation. As shown in Fig 3B, the presence of genistein reduced dramatically biofilm formation ability in the wild-type but not in the *nodD1* mutant strain. The HH103 *nolR* derivative formed less amount of biofilm than the wild-type strain in the absence of flavonoids, and was also negatively affected by the presence of genistein. However, the decrease in biofilm formation ability exhibited by this mutant was not as dramatic as the reduction in EPS production. This result might be due to the fact that NolR is a global regulator, so that its absence might affect other traits involved in bacterial attachment. The biofilm formation ability of the HH103 *nodA* derivative was similar to that of the parental strain, both in the absence or presence of genistein.

### NMR experiments confirm that genistein diminishes *S*. *fredii* HH103 EPS production in a NodD1-dependent manner

**Fig 4:**
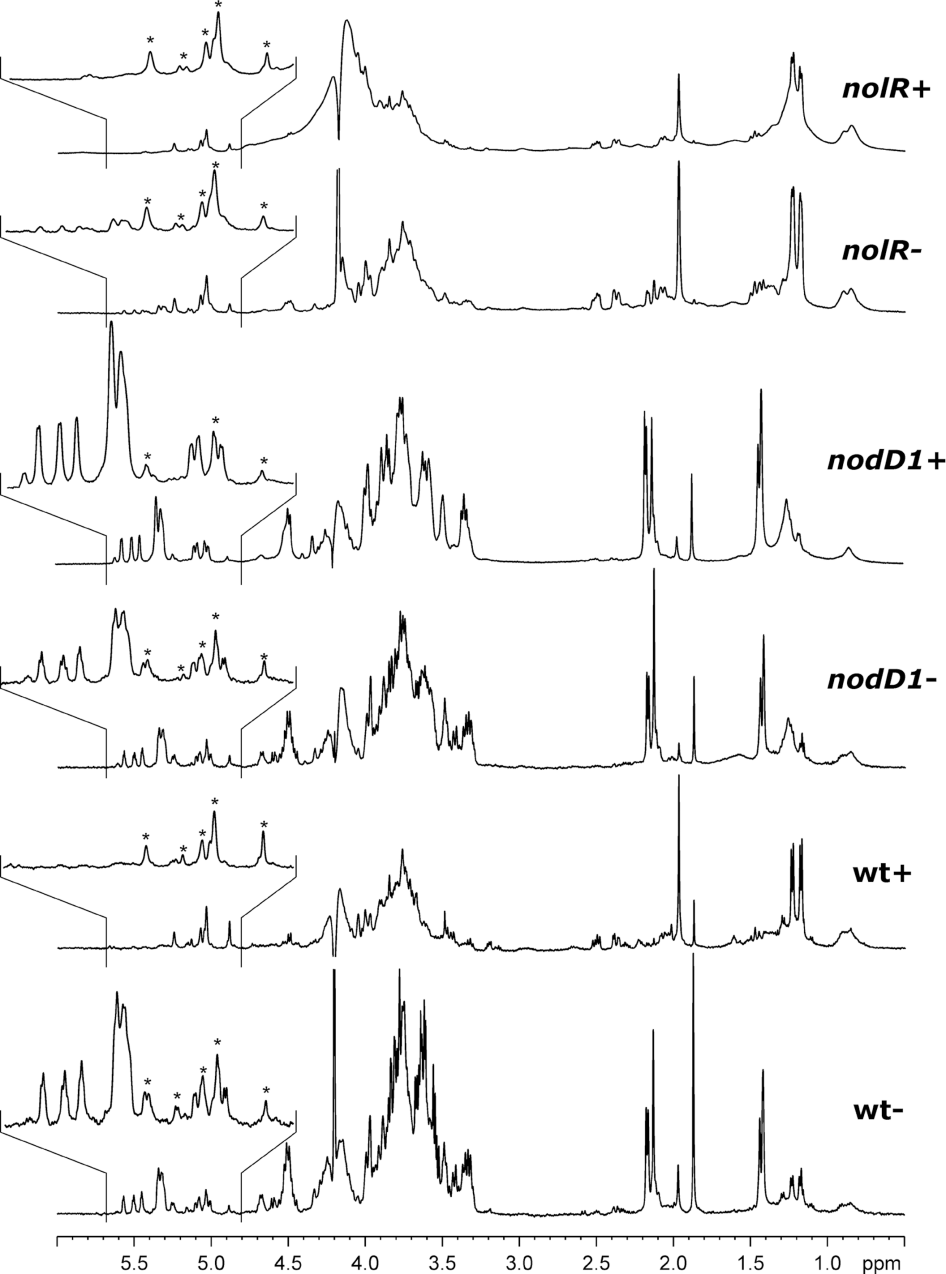
^1^H-NMR (500.20 MHz, 353 K) of the polysaccharides isolated from YMB culture supernatants of *S*. *fredii* HH103 (wt) and its *nodD1* and *nolR* derivatives grown in the absence (-) or presence of genistein (+). Peaks marked with asterisks correspond to a mannan present in yeast extract, one of the components of YMB medium.

Polysaccharides present in culture media were isolated and their ^1^H-NMR spectra acquired. Fig 4 shows that when HH103 is grown in the absence of genistein (wt-), the isolate contains mainly its exopolysaccharide [12], together with minor components such as a mannan from yeast extract or small amounts of KPS [37]. Its *nodD1* derivative has the same behavior, either in the presence or the absence of genistein **(***nodD1*- and *nodD1*+, respectively). Spectra from genistein-induced cultures of HH103 (wt+) and its *nolR* derivative (*nolR*+), on the contrary, do not show signals from EPS. These signals are very weak in non-induced cultures of HH103 *nolR* (*nolR*-). In these cases, ^1^H-NMR spectra present mainly signals from mannan and KPS, indicating that the amounts of EPS have been drastically reduced.

Thus, NMR analyses confirmed that genistein reduces *S*. *fredii* HH103 production of EPS only when a functional copy of *nodD1* is present, and that the absence of NolR also provokes a diminution of the amount of EPS produced. Altogether, our results indicate that in HH103 the production of EPS is regulated in an opposite manner to that of Nod factors and Nops (Nodulation Outer Proteins): it is repressed by NodD1 and flavonoids, which are required for the expression of genes involved in Nod factors and Nops production, but enhanced by the regulatory protein NolR, which in turns represses the expression of *nod* and *nop* genes [6].

### Genistein promotes a reduction of the expression of *exoK* and *exoY2* in *S*. *fredii* HH103 stationary phase cultures

The observed genistein-mediated repression of EPS production by *S*. *fredii* HH103 prompted us to investigate whether the presence of this flavonoid may have an effect on the expression of genes related to EPS production in this strain. Very recently, we have carried out a transcriptomic analysis of the effect of genistein on global gene expression in *S*. *fredii* HH103 [data are available at http://www.ncbi.nlm.nih.gov/bioproject/?term=PRJNA313151]. These studies, which were carried out upon a 24 h genistein treatment (early-stationary phase), did not revealed significant changes in the expression level of *exo* and *exs* genes (│fold changes│≤ 1.6), which are those involved in the production of EPS [12, 17].

Since *S*. *fredii* HH103 production of EPS is only evident in late-stationary phase, we decided to investigate the level of expression of several *exo* genes upon 96 hours of genistein treatment. The organization of the *exo* and *exs* genes of *S*. *fredii* HH103, which are clustered in a region of the largest plasmid (pSfHH103e) has been recently described [12, 17]. For our studies, we selected the *exoA*, *exoY2*, and *exoK* genes. Although biochemical studies of the enzymatic activities of the products encoded by these genes have not been carried out in *S*. *fredii* HH103, their high level of identity with the corresponding *S*. *meliloti* proteins (whose activities have been established, revised in [11]) clearly suggest that they most probably have the same role. Thus, ExoA would be involved in the addition of the first glucose residue to the lipid-galactose structure of the nascent EPS repeating unit, ExoY could participate in the addition of the first sugar to the lipid carrier, and ExoK could act as a glucanase involved in the cleavage of high- (HMW) to low-molecular-weight form of EPS.

**Fig 5:**
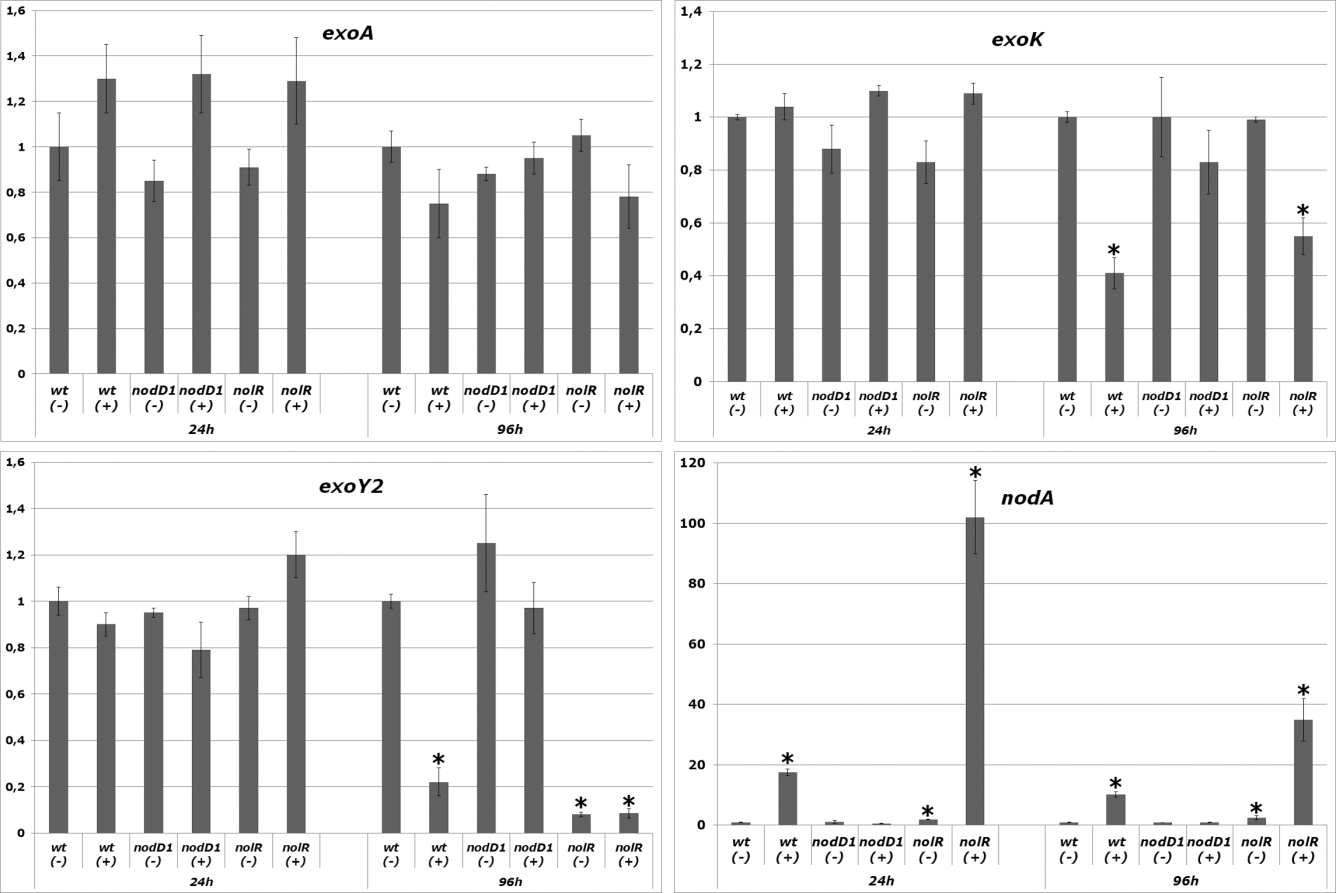
*q*RT-PCR analysis of the effect of genistein in the expression level of *exoA*, *exoK* and *exoY2* expression in *S*. *fredii* HH103 and its *nodD1* and *nolR* mutant derivatives in early (24 h) and late (96 h) stationary phase cultures. (-) and (+) denote the absence and presence of genistein respectively. The presence of an asterisk denotes a significant difference at the 1% level with respect to the expression level in the wild type strain grown in the absence of genistein.

For the analysis of *exo* gene expression, quantitative RT-PCR (*q*PCR) were done by using cDNA from early (24 hours) or late (96 hours) stationary phase cultures of *S*. *fredii* HH103 and its *nodD1* and *nolR* derivatives grown in the presence or absence of genistein (Fig 5). In addition to *exoA*, *exoK* and *exoY2*, the *nodA* gene was also studied as a control of a gene induced by NodD1 and flavonoids and repressed by NolR. As expected, the expression of *nodA* was strongly induced by genistein when a functional NodD1 protein was present (wild-type), being this effect enhanced by the absence of NolR (*nolR* mutant background). After 24 hours of growth, the levels of expression of *exoA*, *exoK* and *exoY2* in the *nodD1* and *nolR* mutant backgrounds were not different from those of the wild-type strain, both in the absence or presence of genistein. Upon 96-hours of genistein treatment, the expression of *exoA* was neither affected by the presence of flavonoids in the three strains analyzed. In contrast, both *exoK* and *exoY2* showed reduced expression when both NodD1 and genistein were present (induced cultures of the wild-type and *nolR* mutant strains). This effect was stronger in the case of *exoY2*, whose expression level was 5-fold and 10-fold reduced in HH103 and in the *nolR* mutant background respectively. In the case of the *nolR* mutant, this reduction in the expression level of *exoY2* was observed both in the presence and absence of genistein.

## Discussion

The rhizobia-legume nitrogen-fixing symbiosis is based on the interchange of signals between both symbionts [2–4]. It is well established that flavonoids exuded by legume roots interact with the bacterial NodD protein inducing the expression of *nod* genes, which are responsible for the production and secretion of bacterial Nod factors. These molecular signals, in turns, elicit root hair curling and the formation of nodule primordia and are also required for infection thread formation. In some rhizobia, including *S*. *fredii* strains, flavonoids and NodD also induce the expression of *ttsI*, which codes for the transcriptional activator of a symbiotic type III secretion system responsible for the delivery of bacterial effector proteins into host plant cells (revised in [8]).

In *S*. *fredii* NGR234, flavonoids are known to also influence surface polysaccharides production. Thus, the presence of apigenin (an effective *nod* gene inducer for this strain) reduces the amount of K-antigen capsular polysaccharide produced in a process mediated by *nodD1* and *y4gM* [15] and leads to the production of a rhamnose-rich LPS through the induction of the *fixF*, *rgpF* and *wbgA* genes [16]. None of these changes takes place in *S*. *fredii* HH103, since *y4gM*, *fixF*, *rgpF*, and *wbgA* are not present in this strain [17].

In this work, we report that *nod* gene inducing flavonoids such as genistein reduces HH103 EPS production in a NodD1-dependent manner. To our knowledge, this is the first time that this mechanism of EPS repression is reported in rhizobia. Previous works showed that in *S*. *fredii* USDA193, genistein provoked changes in EPS production [18], but these changes were structural (lower average molecular mass and reduced uronic acids contents compared to control) and, although they were pSym-dependent, it has not been studied whether they were mediated by NodD proteins. In *S*. *fredii* USDA191 the presence of extra copies of *nodD2* leaded to rough colony morphology whereas extra copies of *nodD1* only reduced very slightly production of EPS in this strain [38, 39]. In addition these changes did not depend on the presence of flavonoids. In *S*. *fredii* HH103, the absence of *nodD1* but not that of *nodD2* led to a mucoid phenotype in the presence of flavonoids, indicating that the repressor effect of flavonoids in this strain is mediated by NodD1. However, the presence of plasmid pMUS746 in the wild-type strain also reduced its mucoidy in the presence of flavonoids, indicating that, when overexpressed, NodD2 is also able to repress EPS production in HH103. The recent sequencing of the *S*. *fredii* HH103 genome has revealed the presence of 15 putative *nod* boxes [17], but none of them is located upstream of genes directly involved in the biosynthesis of EPS (*exo* and *exs* genes). Therefore, the effect of NodD1 and genistein on *S*. *fredii* HH103 EPS production most probably is mediated by other regulatory proteins which remain to be identified.

In contrast to the results presented in this work, in both *S*. *meliloti* Rm1021 and *Rhizobium leguminosarum* bv. *trifolii* 24.2, the presence of flavonoids has been shown to enhance EPS production [34, 40]. In Rm1021 this effect is mediated by the transcriptional regulator SyrM, which is connected to the *nod* regulon through NodD3 and that stimulates EPS production through SyrA [41, 42]. In *S*. *fredii* strains *syrM* is present, but both *nodD3* and *syrA* are missing [17, 43]. *R*. *leguminosarum* bv. *trifolii* lacks a copy of *syrM*, and in strain 24.2 the effect of flavonoids contained in clover root exudates on EPS production is exerted though the RosR transcriptional regulator [40]. In *S*. *fredii* HH103 there are two orthologues of RosR, called MucR1 and MucR2 [17, 43]. We are currently analyzing the role of the *S*. *fredii* HH103 *syrM*, *mucR1*, and *mucR2* genes of the effect of flavonoids on EPS production in this strain. Our preliminary results show that HH103 *mucR1* is an activator of EPS production, which is in agreement with recent results obtained for another *S*. *fredii* strain, CCBAU45436 [44], but it is not connected with the effect exerted by flavonoids. With respect to *S*. *fredii* HH103 *syrM*, most probably transcription of this gene is dependent on *nod* box 19 and activated by NodD1 and genistein [17], what would make this gene a good candidate for EPS regulation. Another HH103 gene that could be involved in EPS regulation is pSfHH103d_161 [17], whose expression is most probably driven by *nod* box 10. This gene codes for a putative protein that is 58% identical to *R*. *leguminosarum* biovar *phaseoli* PsiB, an EPS repressor which has not been studied in other rhizobia [45]. Further research is required to elucidate whether *syrM* and/or pSfHH103d_161 could be involved in the genistein induced repression of EPS production. In any case, results presented in this paper as well as previous results of our group [6] demonstrate that this effect is exerted through the *nod* regulon and that it involves NodD1 and NolR as a repressor and an activator respectively of EPS production, indicating that in *S*. *fredii* HH103 EPS is regulated in just the opposite way that Nod factors and type 3 secreted effectors, which are induced by NodD1 and flavonoids and repressed by NolR [6, 21, 25]. Regardless what other genes participate in this regulatory network, the presence of genistein and/or the lack of NolR led to a significant decrease of the expression of the HH103 *exoK* and *exoY2* genes as demonstrated in this work by quantitative RT-PCR. Our results are in agreement with previous observations carried out in *S*. *meliloti and R*. *leguminosarum* bv. *trifolii*, in which modulation of EPS production is achieved by regulating the expression of a small number of *exo*/*pss* genes [46, 47]. Remarkably, in the case of *S*. *meliloti* two of these genes are *exoK* and *exoY* [46].

Another interesting finding found in this study is the fact that the *nodA* mutant derivative of *S*. *fredii* HH103, when compared to the parental strain, showed alterations in its appearance when grown on YMA plates as well as in the amount of glucose equivalents found in the extracellular milieu in the presence of genistein. Elucidating the nature of these alterations is an issue that we will address in the next future. Remarkably, inactivation of *nodA*, which results in inability to produce Nod factors, does not alter the HH103 ability to attach onto plastic surfaces. In contrast, in *S*. *meliloti* Nod factors are required for the establishment of the three-dimensional architecture of biofilms [48], which suggests that different sinorhizobial strains might have developed different strategies for biofilm formation.

EPS plays an essential role in nodule invasion in symbiosis with legumes forming indeterminate nodules, such as the interactions *S*. *meliloti*-*Medicago* or *Rhizobium leguminosarum* bv. *trifolii*-*Trifolium* [11 and references therein]. On the other hand, although traditionally EPS has been considered not essential in symbiosis forming determinate nodules, recent works show that production of altered forms of this polysaccharide can lead to impairments in the interactions *Bradyrhizobium japonicum* USDA110-soybean and *Mesorhizobium loti*-*Lotus japonicus* [49, 50]. These results can be explained by the recent discovery of a *Lotus* LysM receptor which senses the presence of EPS and which is able to block root infection when non-appropriate forms of this polysaccharide are detected [51].

So, an open question is why *S*. *fredii* HH103 EPS production is repressed by flavonoids present in legume root exudates? *S*. *fredii* HH103 has a broad host range of nodulation [52], which allows studying the role of its molecular signals with different host legumes. Regarding surface polysaccharides, only CG has been proven to be essential for nodulating all its host plants tested so far [53]. In previous works, we have analyzed the symbiotic performance of an EPS deficient derivative of this strain due to an *exoA* mutation [10, 13, 14]. This mutant was able to induce nitrogen fixing nodules in the different legumes analyzed regardless they formed indeterminate (*Glycyrrhiza uralensis and Cajanus cajan*) or determinate (*Glycyne max and Vigna unguiculata*) nodules, suggesting a low importance for this polysaccharide in HH103 symbiotic ability. In fact, with soybean (*G*. *max*) the HH103 *exoA* derivative not only showed a slightly increased symbiotic performance but it was even more competitive for nodulating this plant [12, 13], which suggest that, at least with this legume, the presence of EPS can have a slight but detrimental effect on symbiosis. However, this would not be always the case, since opposite results were found with cowpea (*V*. *unguiculata*), in which the *exoA* derivative of *S*. *fredii* HH103 showed slightly decreased symbiotic performance and lower competitiveness ability than the parental strain [12, 13].

In summary, we show that in *S*. *fredii* HH103 *nod* gene inducing flavonoids, in addition to promote the production of Nod factors and the delivery of type 3 secreted effectors, modify bacterial surface by repressing EPS production. This change is different to those described in other rhizobia such as *S*. *fredii* NGR234, in which flavonoids repress KPS and induce a novel type of LPS, or *S*. *meliloti* and *R*. *leguminosarum* bv. *trifolii*, which increase EPS production upon treatment with flavonoids. Thus, this study represents a new example of that mechanisms found in model bacteria are not always present in other rhizobial strains.

## Supporting Information

S1 Table Primers used in quantitative RT-PCR (*q*PCR) experiments. (DOC)
