# Supplementary material for: Exopolysaccharide Production by Sinorhizobium fredii HH103 Is Repressed by Genistein in a NodD1-Dependent Manner
Source: PLoS One. 2016 Aug 3;11(8):e0160499. doi: 10.1371/journal.pone.0160499 (PMC4972438; doi:10.1371/journal.pone.0160499)
Supplement: S1 Table — (DOC) [file pone.0160499.s001.doc]

**S1 Table.** Primers used in quantitative RT-PCR (*q*PCR) experiments.

| **Primer** | **Sequence (5´🡪3´)** | **Predicted length of PCR products (bp)** |
| --- | --- | --- |
| rt-16S-F2 | GATACCCTGGTAGTCCAC | 167 |
| rt16S-R2 | TAAACCACATGCTCCACC |  |
| rt-exoA-F2 | CATCTGGATGACCGACAG | 113 |
| rt-exoA-R2 | GCTTGAGGATGTTCTTCG |  |
| rt-exoK-F | CGATTTCGAGGTGCTTGG | 144 |
| rt-exoK-R | TCCTTCTCCCAGACGAAG |  |
| exoY2rt-F | ATCGGGTACTGCAGGAAT | 158 |
| exoY2rt-R | GCGAATGATGTTGAGGAG |  |
| qnodA-F | CGTCATGTATCCGGTGCTGCA | 172 |
| qnodA-R | CGTTGGCGGCAGGTTGAGA |  |
